# Augmented B Lymphocyte Response to Antigen in the Absence of Antigen-Induced B Lymphocyte Signaling in an IgG-Transgenic Mouse Line

**DOI:** 10.1371/journal.pone.0008815

**Published:** 2010-01-21

**Authors:** Rong-Yong Man, Taishi Onodera, Emi Komatsu, Takeshi Tsubata

**Affiliations:** 1 Laboratory of Immunology, Graduate School of Biomedical Sciences, Tokyo Medical and Dental University, Tokyo, Japan; 2 Department of Immunology, Medical Research Institute, Tokyo Medical and Dental University, Tokyo, Japan; 3 Core Research for Evolutional Science and Technology, Japan Science and Technology Agency, Kawaguchi, Japan; University of Miami, United States of America

## Abstract

IgG-containing B cell antigen receptor (IgG-BCR), the BCR mostly expressed on memory B cells, contains a distinct signaling function from IgM-BCR or IgD-BCR expressed on naïve B cells. Because naïve B cells transgenic for IgG exhibit augmented response to antigens similar to memory B cells, the distinct signaling function of IgG-BCR appears to play a role in augmented antibody responses of memory B cells. However, how IgG-BCR signaling augments B cell responses is not yet well understood. Here we demonstrate that B cells from IgG-transgenic mice are anergic with defect in generation of BCR signaling upon BCR ligation. However, these IgG-transgenic B cells generate markedly augmented antibody response to a T cell-dependent antigen, probably due to hyper-responsiveness to a T cell-derived signal through CD40. Both BCR signaling defect and augmented response to CD40 ligation are partially restored in xid IgG-transgenic mice in which BCR signaling is down-modulated due to a loss-of-function mutation in the tyrosine kinase Btk crucial for BCR signaling. Thus, IgG-BCR induces augmented B cell responses in the absence of antigen-induced BCR signaling probably through high ligand-independent BCR signaling that may “idle” B cells to make them ready to respond to T cell help. This finding strongly suggests a crucial role of ligand-independent signaling in receptor function.

## Introduction

The B cell antigen receptor (BCR), composed of the membrane form of immunoglobulin and Igα/Igβ, plays a crucial role in both development and homeostasis of B cells as well as their responses to antigen stimulation [1]. BCR signaling generated in the absence of antigen, its ligand, is known as tonic BCR signaling, and is essential for maintenance of B cell population by inducing survival of B cells [2], [3]. Moreover, signal strength of the tonic BCR signaling appears to regulate whether immature B cells differentiate to either one of the two major subsets of conventional B cells, i.e., follicular B cells and marginal zone B cells [1], [4]. B cells deficient in BCR signal components such as Btk favor marginal zone B cell fate whereas those deficient in negative regulators of BCR such as CD22 favor follicular B cell fate, suggesting that differentiation to follicular B cells requires high tonic BCR signal strength, whereas low tonic BCR signaling induces differentiation to MZ B cells. In contrast, the number of MZ B cells is increased in some autoantibody-transgenic mouse lines carrying mostly self-reactive B cells [5]–[7], suggesting that continuous BCR ligation by interaction with self-antigens may also induces differentiation to MZ B cells.

Continuous BCR signaling generated by interaction with self-antigens during B cell development causes B cell anergy, in which B cells are no longer activated for proliferation and differentiation to plasma cells after antigen stimulation [8]. Silencing self-reactive B cells by inducing anergy may play a role in maintenance of self-tolerance. Anergic B cells characteristically exhibit reduced BCR expression on the surface [8], [9] and augmented expression of molecules such as CD44 [8], [10], Fas [11], [12] and CD93 [13]. Recently, Merrel et al. [13] demonstrated that anergic B cells are accumulated in B220^+^CD93^+^IgM^lo^CD23^+^ T3 B cells. Ex vivo analysis of anergic B cells has demonstrated that baseline activation of BCR signaling molecules is augmented whereas BCR ligation generates only a weak signaling [8], [14], suggesting that ligation-induced BCR signaling is perturbed probably due to continuous BCR signaling in vivo.

Among the five distinct classes of immunoglobulins, IgG-containing BCR (IgG-BCR), the BCR mostly expressed on memory B cells, exhibits a distinct signaling function from IgM-BCR or IgD-BCR [15]–[17], both of which are expressed on naïve B cells. Transgenic B cells expressing IgG or chimeric IgM/G containing the extracellular region of IgM and cytoplasmic tail of IgG exhibit augmented antibody production after primary antigen stimulation [16]. This finding indicates that expression of IgG instead of IgM or IgD augments antibody response in naïve B cells probably by augmented signaling through IgG-BCR, and that the cytoplasmic tail of IgG plays a role in augmented BCR signaling. As memory B cells mostly express IgG-BCR, augmented antibody production during memory responses may involve augmented signaling function of IgG-BCR. In B cell lines, ligation of IgG-BCR induces stronger signaling including Ca^2+^ mobilization and phosphorylation of ERK than ligation of IgM or IgD does [15], [18]. Recent studies have demonstrated that increased Ca^2+^ mobilization is also induced by BCR ligation in transgenic B cells expressing IgG or IgM/G [17], [19]. In these transgenic B cells, however, both phosphorylation of signaling molecules such as ERK and expression of BCR-induced genes induced by BCR ligation are rather diminished compared to IgM-expressing B cells. Thus, the model in which augmented signaling induced by IgG-BCR ligation causes augmented antibody production is no longer valid.

In this study, we established mice transgenic for IgG reactive to the hapten (4-hydroxy-3-nitrophenyl)acetyl (NP) to address how IgG-BCR induces augmented antibody responses. Although the number of B cells is markedly reduced, antibody production of IgG-transgenic (IgG-tg) B cells was markedly augmented after immunization with a T cell-dependent protein antigen, in agreement with previous observations [16], [20]–[22]. Surprisingly, these B cells did not generate signaling after BCR ligation in vitro, but showed augmented proliferation upon ligation of CD40, the receptor of the T cell-derived co-stimulatory signal CD40L (CD154). IgG-tg B cells exhibited increased basal signaling, suggesting the presence of high tonic signaling through IgG-BCR, and defects in B cell generation and BCR signaling were partially restored in IgG-tg mice carrying the loss-of-function xid mutation in the BCR signaling molecule Btk probably through reduction of tonic BCR signaling. These results suggest that high tonic BCR signaling causes both decrease in the B cell number and BCR signaling defect. Moreover, high tonic IgG-BCR signaling may cause hyper-proliferation upon CD40 ligation as CD40 signaling synergistically induces B cell proliferation with BCR signaling. Thus, IgG-BCR generates high tonic signaling thereby generating augmented antibody responses to T cell-dependent antigens in the absence of antigen-induced BCR signaling probably by augmented proliferative responses to the T cell-derived signal CD40L.

## Results

### B Cells Expressing IgG-BCR Exhibit an Anergic Phenotype

**Figure 1:**
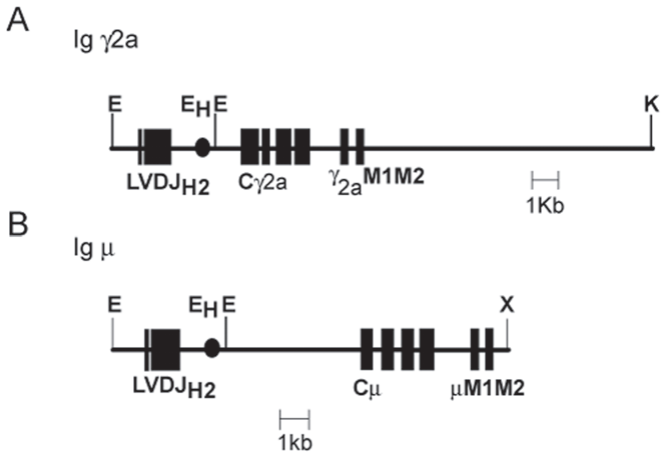
Schematic representation of Ig γ2a and μ H chain constructs. Wide boxes indicate protein coding regions, and filled circle represents the μ intron enhancer(E_H_). E, EcoRI; K, KpnI; X, XhoI.

**Figure 2:**
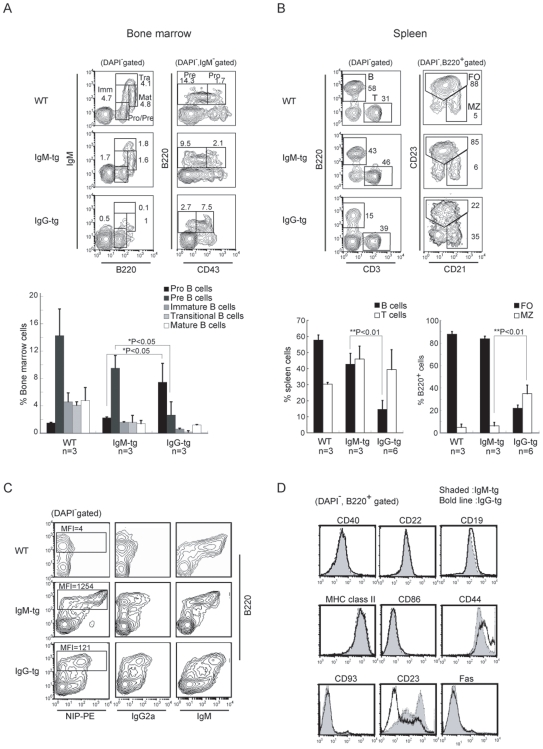
Flow cytometry analysis of bone marrow and spleen cells from wild type (WT) C57BL/6, IgM-tg and IgG-tg mice. (A) Analysis of bone marrow cells. Cells were stained for B220, IgM, and CD43. Percentages of pro B cells (IgM^−^, B220^+^, CD43^+^), pre B cells (IgM^−^, B220^+^, CD43^−^), immature B cells (B220^lo^, IgM^med^), transitional B cells (B220^+^, IgM^hi^), and mature B cells (B220^hi^, IgM^med^) are calculated. Numbers of mice analyzed are indicated, and data are shown as mean (±SD) (bottom). (B) Subsets of spleen B cells. Spleen cells were stained for CD3, B220, CD21 and CD23. Percentages of T cells and B cells (left panel) in total spleen cells, and those of MZ B cells (B220^+^, CD21^hi^, CD23^lo/−^) and follicular B cells (B220^+^, CD21^inter^, CD23^hi^) in total B cells (right panel) are calculated. Numbers of mice analyzed are indicated, and data are shown as mean (±SD) (bottom). (C) Expression of BCR on spleen B cells. Spleen cells were stained for NIP binding, IgG2a, IgM and B220. Mean fluorescence intensity (MFI) of NIP binding is indicated. (D) Expression of various molecules on spleen B cells. Spleen cells from IgG-tg (open histograms) and IgM-tg (shaded histograms) mice were analyzed for expression of CD40, CD22, CD19, MHC class II, CD86, CD44, CD23, Fas, and CD93. All histograms are gated on B220^+^ cells.

To address the functional properties of IgG-BCR, we generated transgenic mice expressing anti-NP Ig γ2a chain (Figure 1a), and crossed these mice with Ig κ chain-deficient mice, resulting in IgG-tg mice in which almost all the B cells express NP-reactive IgG2a because of its expression of both VH186.2-containing anti-NP Ig γ2a chain and λ L chain [23], [24]. We established two independent Ig γ2a chain-transgenic mouse lines, and used one of them for this study, but the other line shows basically the same phenotype. As a control, we generated anti-NP IgM-tg mice that express both VH186.2-containing Ig μ chain (Figure 1b) and λ L chain in almost all the B cells. First, we analyzed B cell development in the bone marrow and spleen of IgM-tg and IgG-tg mice by flow cytometry. In IgG-tg bone marrow, the percentage of B220^+^CD43^+^ pro B cells was markedly increased whereas that of B220^+^CD43^−^ pre B cells was decreased (Figure 2a), suggesting that maturation of pro B to pre B cells is perturbed. The percentage of B220^+^ B cells in the spleen was markedly reduced (Figure 2b), probably due to poor B cell production in the bone marrow. Analysis of spleen B cells demonstrated both increase in the percentage of B220^+^CD21^hi^CD23^lo/−^ marginal zone (MZ) B cells and decrease in B220^+^CD21^inter^CD23^+^ follicular B cells. Thus, expression of IgG induced severe maturational block from pro B to pre B cells, and biased differentiation to MZ B cells over follicular B cells.

When we analyzed phenotype of spleen B cells, binding to the antigen NIP was 10 times lower in IgG-tg B cells than that in IgM-tg B cells, indicating that BCR expression is down-modulated in IgG-tg B cells (Figure 2c). IgG-tg B cells exhibited increased expression of CD44 and decreased expression of CD23, whereas expression of other molecules including CD19, CD22, CD40, CD86, CD93, CD95 and MHC class II was not altered compared to IgM-tg B cells (Figure 2d). Because reduced expression of BCR and CD23, and increased expression of CD44 were shown in anergic B cells, these results indicated that IgG-tg B cells show anergic phenotype.

### IgG-tg B Cells Do Not Respond to BCR Ligation but Generate Augmented Response to Co-Stimulation

**Figure 3:**
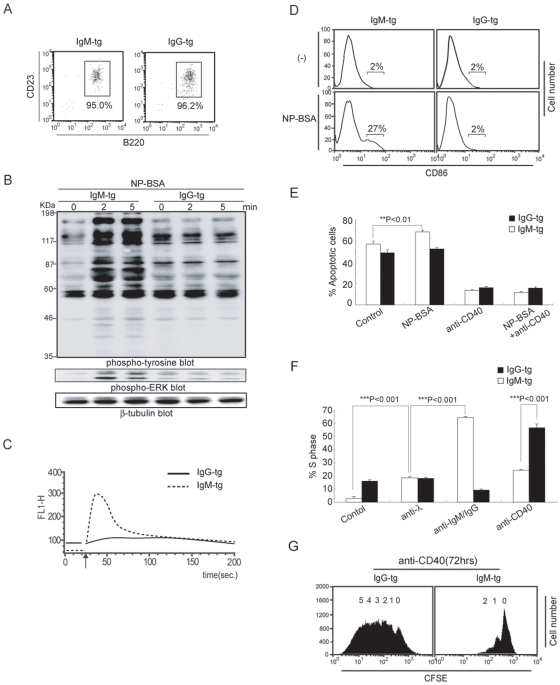
IgG-tg B cells generate an augmented proliferative response to CD40 ligation but no antigen-induced BCR signaling. CD23^+^ follicular B cells were isolated from IgM-tg and IgG-tg spleen by magnet sorting. (A) Purity of CD23^+^ follicular B cells used for in vitro analysis. Expression of B220 and CD23 was analyzed by flow cytometry. Percentages of B220^+^CD23^+^ follicular B cells are indicated. (B and C) Antigen-induced BCR signaling. Purified CD23^+^ follicular B cells were stimulated with 0.2 µg/ml NP_15_-BSA for 2 and 5 min. Phosphorylation of cellular substrates in total cell lysates (B, upper panel) and that of ERK (B, middle panel) were analyzed by Western blotting. Western blot analysis of β-tubulin was done as a loading control (B, lower panel). Alternatively, cells were loaded with Fluo-4/AM, and intracellular free Ca^2+^ was measured by flow cytometry (C). Cells were added with 0.2 µg/ml NP_15_-BSA at 25s as indicated by arrows, and measurement of free Ca^2+^ was continued for 200s. Representative data of three experiments are shown. (D) Expression of CD86. Purified CD23^+^ follicular B cells were cultured with or without 0.2 µg/ml NP_15_-BSA for 48 hrs, and analyzed for expression of CD86 by flow cytometry. Percentage of CD86^+^ cells is indicated. (E) Apoptosis of B cells. Purified CD23^+^ follicular B cells were cultured with indicated reagents for 24 hrs. Percentages of apoptotic cells with hypodiploid DNA were measured by flow cytometry. Data are shown as average of three experiments (±SD). (F) Cell cycle progression of B cells. Purified CD23^+^ follicular B cells were cultured with indicated reagents for 48 hrs, and were pulsed with BrdU for the last 20 min of the culture. Percentages of BrdU-incorporated cells (% S phase) were measured by flow cytometry. Data are shown as average of three experiments (±SD). (G) Division of B cells. Purified CD23^+^ follicular B cells were labeled with CFSE, and cultured with anti-CD40 antibody for 72 hrs. Cells were analyzed by flow cytometry for CFSE labeling. The numbers of cell division are indicated.

To ask the signaling function of IgG-BCR, we purified CD23^+^ follicular B cells from IgM-tg and IgG-tg spleen (Figure 3a), and stimulated with the antigen NP-BSA. After antigen stimulation, various cellular substrates including ERK were tyrosine-phosphorylated, and both intracellular Ca^2+^ concentration and CD86 expression were increased in IgM-tg B cells (Figure 3b–d). The basal level of substrate phosphorylation and intracellular Ca^2+^ concentration in unstimulated IgG-tg B cells were higher than that in IgM-tg B cells (Figure 3b–c), indicating high tonic signaling through IgG-BCR. However, antigen stimulation did not increase either substrate phosphorylation, intracellular Ca^2+^ concentration or CD86 expression in IgG-tg B cells (Figure 3b–d), indicating that BCR ligation does not generate signaling in these B cells. As continuous BCR signaling generated by interaction with self-antigen causes B cell anergy including signal defect upon ex vivo BCR ligation [8], [14], high tonic signaling through IgG-BCR may be involved in BCR signal defect in IgG-tg B cells.

B cells undergo proliferation when BCR is ligated by anti-Ig antibodies. In contrast, BCR ligation by antigen induces apoptosis, and BCR-ligated B cells require co-stimulatory signaling through CD40 for activation and proliferation [25]. To examine in vitro B cell response to BCR ligation and co-stimulation, we cultured purified follicular B cells with various stimuli including antigen, anti-Ig antibodies and anti-CD40 antibody. Both anti-IgM and anti-λ antibodies reduced apoptosis and induced cell cycle progression to the S phase in IgM-tg B cells (Figure 3e–g), although anti-IgM antibody induced survival and cell cycle progression more efficiently than anti-λ antibody did. In contrast, anti-IgG antibody or anti-λ antibody did not induce survival or cell cycle progression in IgG-tg B cells, supporting the notion that BCR ligation does not generate signaling in IgG-tg B cells. However, when we stimulated B cells with anti-CD40 Ab, IgG-tg B cells progressed into the cell cycle more efficiently than IgM-tg B cells did (Figure 3e–g). Both reduced proliferative response to BCR ligation and augmented response to CD40 ligation exhibited by IgG-tg follicular B cells are shared by MZB cells from normal mice [26]–[28]. However, these functional properties of MZB cells may not contribute to the altered response of IgG-tg B cells as we used highly purified follicular B cells for this study (Figure 3a). This notion is further supported by our finding that IgG-tg B cells do not generate signaling upon BCR ligation whereas MZB cells exhibit rather augmented BCR ligation-induced signaling [26], [29]. As CD40 signaling activates B cells synergistically with BCR signaling, IgG-tg follicular B cells efficiently undergo cell cycling by stimulation with anti-CD40 antibody probably due to augmented tonic BCR signaling.

### Augmented Antibody Production in IgG-tg Mice

**Figure 4:**
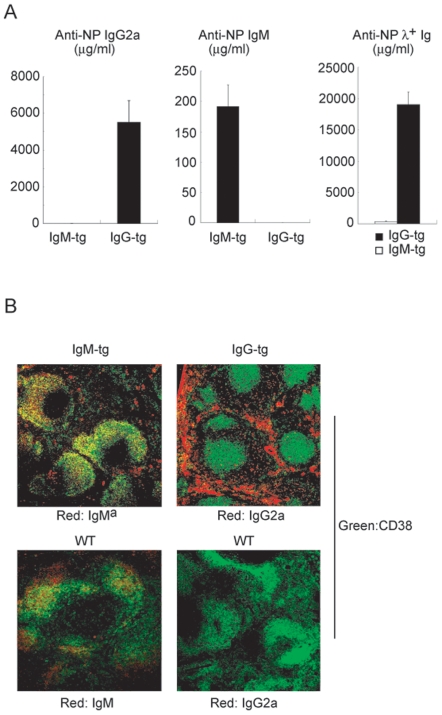
Augmented antibody production in unimmunized IgG-tg mice. (A). Serum levels of anti-NP IgM, IgG2a, and λ L chain-containing Ig. Sera from 13-wk-old IgM-tg and IgG-tg mice were analyzed by ELISA. Data are shown as mean ± SD. (B). Histological analysis. Frozen spleen sections from upper IgM-tg (left panel), IgG-tg (right upper panel) and wild type (WT) C57BL/6 (lower panes) mice were stained either anti-IgM^a^ Ab (left upper panel), anti-IgG2a Ab (right upper panel) (red), anti-IgM Ab (left lower panel) or anti-IgG2a (right lower panel) together with anti-CD38 Ab (green).

To address Ab production in IgM-tg and IgG-tg mice, we first measured serum levels of Ig transgene-derived Ab in untreated mice. The serum concentration of anti-NP Ab in IgG-tg was almost 100 times higher than that in IgM-tg mice (Figure 4a). When we histologically analyzed spleen sections, a large number of IgG^+^ plasma cells were accumulated in the red pulp in unimmunized IgG-tg mice (Figure 4b). This result indicated that B cells undergo spontaneous maturation to plasma cells in these mice.

**Figure 5:**
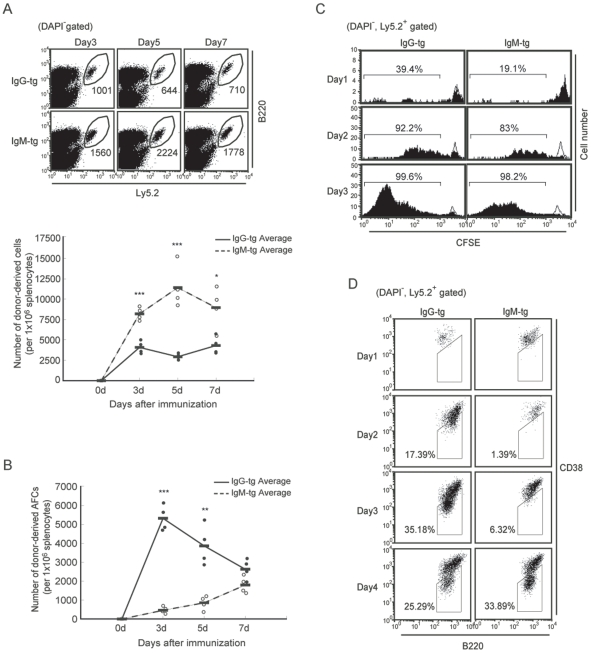
IgG-tg B cells rapidly differentiate to AFCs and GC B cells after immunization with a T cell-dependent antigen. Splenic CD23^+^ follicular B cells were isolated from IgM-tg and IgG-tg mice by magnet sorting as indicated in the legend of Figure 2. Either untreated (A, B and D) or CFSE-labeled (C) follicular B cells (4×10^5^) were injected intravenously into CGG-primed B6.Ly5.1 mice. One day later, recipients were immunized intraperitoneally with 50 µg NP_25_-CGG in alum. Indicated days after immunization, spleen cells from recipient mice were obtained. (A) Expansion of donor-derived B cells. Cells were stained for B220 and Ly5.2, and analyzed by flow cytometry. Dead cells were excluded by staining with DAPI. Representative flow cytometry plots are shown (upper panel). Numbers of donor-derived B cells (B220^+^, Ly5.2^+^) in 1×10^6^ total spleen cells were calculated (lower panel). Circles represent data from individual recipients, and bars show the mean of each group. Closed circles and solid line, recipients transferred with IgG-tg B cells; Open circles and dotted line, recipients transferred with IgM-tg B cells. Statistical analysis was done by Student's *t*-test (*, *p*<0.05; **, *p*<0.01; ***, *p*<0.001). (B) Generation of donor-derived AFCs. Frequency of AFCs secreting donor-derived Abs in 1×10^6^ total spleen cells from recipients that received IgM-tg B cells or IgG-tg B cells were measured by an ELISPOT assay. Circles and lines are as in the legend to Figure 4A. Statistical analysis was done by Student's *t*-test (*, *p*<0.05; **, *p*<0.01; ***, *p*<0.001). (C) Division of donor-derived B cells in recipients. Dilution of CFSE in Ly5.2^+^ donor-derived cells was analyzed by flow cytometry. The numbers of the cell division are indicated. As a negative control, recipients were i.p. injected with PBS instead of NP-CGG/Alum, and analyzed in parallel (open histogram). Percentages of cells that underwent cell division are indicated. Data are representative of two independent experiments. (D) Generation of donor-derived GC B cells. Cells were stained for B220, CD38 and Ly5.2, and analyzed by flow cytometry. Dead cells were excluded by staining with DAPI. Percentages of donor-derived GC B cells (B220^+^, Ly5.2^+^, CD38^lo^) in 2×10^5^ total spleen cells are indicated. Representative data of three experiments are shown.

Next, we addressed in vivo Ab responses to antigen stimulation. To avoid blocking of antigen by secreted antibodies in IgM and IgG-tg mice, we purified follicular B cells from IgM-tg and IgG-tg spleen, and transferred them to the Ly5.1^+^ recipient mice that do not spontaneously produce anti-NP antibody. Because donor B cells express Ly5.2 and carry the transgene encoding IgM^a^ or IgG2a^a^, donor-derived B cells and Ab can be distinguished from those of recipient mice (Ly5.1^+^, IgM^b^, IgG2a^b^). When the recipient mice were immunized with the T cell-dependent protein antigen NP-CGG, both the number of donor-derived B cells and AFCs generated from donor B cells were increased regardless whether donor B cells were obtained from IgM-tg or IgG-tg mice (Figure 5a–b). IgG-tg B cells generated a much larger number of AFCs than IgM-tg B cells did especially at the early phase of the response, indicating that IgG-tg B cells rapidly mature to plasma cells. In contrast, the increase in the number of IgG-tg B cells was not so extensive as that of IgM-tg B cells after immunization. However, when we transferred CFSE-labeled donor B cells, IgG-tg B cells exhibited more dilution of CFSE than IgM-tg B cells did (Figure 5c), indicating rapid division of IgG-tg B cells compared to IgM-tg B cells. These results suggested that, after immunization, IgG-tg B cells expand more rapidly than IgM-tg B cells but mature to plasma cells more efficiently, resulting in mild reduction in the number of IgG-tg B cells. Moreover, IgG-tg B cells differentiated to B220^+^CD38^lo^ GC B cells more rapidly than IgM-tg B cells (Figure 5d). Taken together, IgG-tg B cells rapidly expand, and differentiate to both GC B cells and plasma cells after immunization with T cell-dependent antigens.

### IgG-tg B Cells Do Not Respond to In Vivo Stimulation with a T Cell-Independent Polysaccharide Antigen

**Figure 6:**
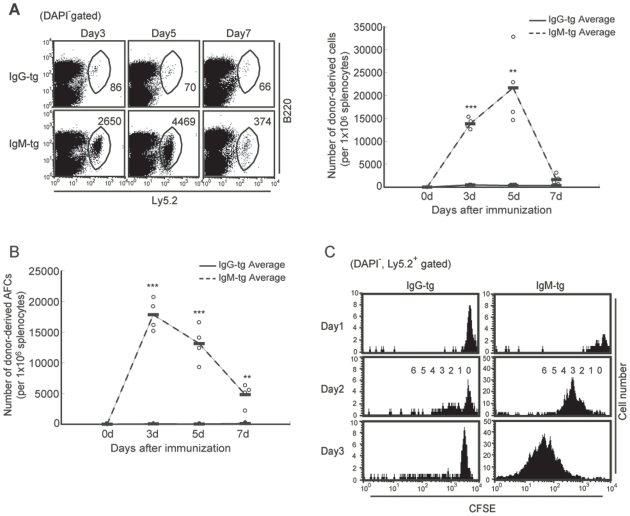
IgG-tg B cells do not respond to a T cell-independent polysaccharide antigen. Splenic CD23^+^ follicular B cells were isolated from IgM-tg mice and IgG-tg mice by magnet sorting as indicated in the legend to Figure 2. Follicular B cells (4×10^5^) were injected intravenously into B6.Ly5.1 mice. One day later, recipients were immunized intravenously with 50 µg NP_50_-Ficoll in PBS. Expansion of donor-derived B cells (A), generation of donor-derived AFCs (B), and cell division of donor-derived B cells in recipients (C) were analyzed as in the legend to Figure 5.

Polysaccharide antigens such as dextran and Ficoll induce T cell-independent antibody production probably because BCR is extensively ligated by highly repetitive structure of these antigens [30]. To address the response of IgG-tg B cells that depends on BCR signaling alone, we transferred IgM-tg and IgG-tg B cells to recipient mice, and immunized them with the T cell-independent antigen NP-Ficoll. After immunization, IgM-tg B cells underwent cell division, expanded and differentiated to AFCs (Figure 6a–c). However, IgG-tg B cells did not show cell division, expansion or differentiation to AFCs at all. Thus, IgG-tg B cells do not respond to in vivo stimulation with the T cell-independent antigen NP-Ficoll probably due to BCR signaling defect.

### The Xid Mutation of Btk Partially Restores B cell Abnormalities in IgG-tg Mice

**Figure 7:**
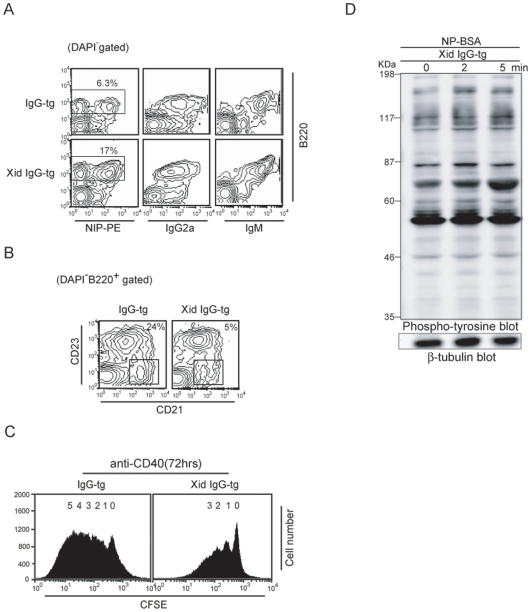
The Xid mutation of Btk partially restores abnormalities of IgG-tg B cells. (A) BCR expression of B cells. Spleen cells from indicated mice were stained for NIP binding, IgG2a, IgM and B220, and analyzed by flow cytometry. Percentages of B cells are indicated. (B) Subsets of spleen B cells. Spleen cells were stained for B220, CD21 and CD23. Percentages of MZ B cells (B220^+^, CD21^hi^, CD23^lo/−^) and follicular B cells (B220^+^, CD21^inter^, CD23^hi^) are indicated. (C) Proliferation of B cells. Purified CD23^+^ follicular B cells were labeled with CFSE, and cultured with anti-CD40 antibody for 72 hrs. Cells were analyzed by flow cytometry for CFSE labeling. The numbers of cell division are indicated. (D) BCR ligation-induced tyrosine phosphorylation of cellular substrates. Purified CD23^+^ follicular B cells were stimulated with 0.2 µg/ml NP_15_-BSA for 2 and 5 min. Phosphorylation of cellular substrates in total cell lysates (upper panel) was analyzed by Western blotting. Western blot analysis of β-tubulin was done as a loading control (lower panel).

To reduce tonic BCR signaling, we crossed IgG-tg mice with xid mice carrying a loss-of-function mutation in the tyrosine kinase Btk crucial for BCR signaling [31], [32]. In xid IgG-tg mice, the number of B cells was markedly increased although the level of BCR expression was not altered (Figure 7a). In contrast, the number of MZ B cells was reduced (Figure 7b), although xid non-transgenic mice show rather increased generation of MZ B cells [33]–[35]. Xid IgG-tg B cells exhibited reduced proliferative response to anti-CD40 antibody (Figure 7c), and BCR ligation-induced tyrosine phosphorylation of cellular substrates was partially restored (Figure 7d). Thus inhibition of tonic BCR signaling by introduction of the xid mutation partially restores abnormalities in IgG-tg mice such as reduced B cell production, increased generation of MZ B cells, augmented B cell response to CD40 ligation and BCR signaling defect. These results suggest that augmented tonic signaling through IgG-BCR plays a role in B cell abnormalities in IgG-tg B cells.

## Discussion

Here we demonstrate that IgG-tg B cells expressing NP-reactive IgG exhibit reduced BCR expression on the cell surface and BCR signaling defect, both of which are characteristically shown in anergic self-reactive B cells [9], [11], [36], [37]. Other phenotypes found in IgG-tg B cells such as increased number of MZ B cells [16], [17], [38], reduced CD23 expression [10] and increased CD44 expression [8], [10] are also found in anergic self-reactive B cells. In contrast, IgM-tg B cells expressing IgM carrying the same antigen specificity as in IgG-tg B cells do not exhibit anergic phenotypes, suggesting that antigen specificity of IgG-tg B cells are not responsible for the anergic phenotypes. Although IgG-tg B cells express endogenous IgM, the anergic phenotype is caused by expression of IgG as IgM expression does not induce such a phenotype. Because anergy is caused by continuous BCR signaling induced by interaction with self-antigens [12], anergy-like phenotypes of IgG-tg B cells suggest continuous signaling through IgG-BCR although it does not appear to interact with self-antigens. It is already well established that continuous low-level BCR signaling known as tonic BCR signaling is generated in normal B cells in the absence of antigen, and plays a crucial role in maintenance of B cell homeostasis [2], [3]. In IgG-tg B cells, efficient signaling capacity of IgG-BCR [15], [18] may cause tonic BCR signaling as strong as BCR signaling generated by antigen stimulation in self-reactive B cells. High tonic BCR signaling in IgG-tg B cells is supported by increased basal activation of signaling molecules compared to IgM-tg B cells, and also accounts for the induction of augmented proliferative response of IgG-tg B cells induced by CD40 ligation. CD40 signaling induces proliferation of B cells synergistically with antigen stimulation. High tonic signaling through IgG-BCR appears to substitute for antigen-induced BCR signaling, and thus CD40 ligation alone may induce efficient proliferation of IgG-tg B cells. Moreover, down-modulation of tonic BCR signaling by introducing the xid mutation of Btk partially restores B cell abnormalities in IgG-tg mice including reduction of B cells, skewed maturation to marginal zone B cells, augmented proliferative response to CD40 ligation and defect of antigen-induced BCR signaling, suggesting that high tonic BCR signaling is responsible for these phenotypes of IgG-tg B cells. Taken together, high tonic BCR signaling appears to induce anergy-like phenotypes but efficient CD40-mediated B cell expansion in IgG-tg B cells.

We demonstrate here that IgG-tg B cells exhibit augmented in vivo response to a protein antigen, in agreement with the previous finding by Martin et al. [16]. Antigen stimulation induces rapid expansion of IgG-tg B cells and their differentiation to plasma cells. However, the antigen stimulation does not generate BCR signaling, indicating that IgG-tg B cells respond to antigen without generating BCR signaling. After antigen stimulation, the antigenic peptide presented by B cells is recognized by specific T helper cells, resulting in antigen-specific interaction of B cells with T helper cells [39], [40]. This interaction may efficiently activate IgG-tg B cells because T cell-derived CD40 signaling alone induces efficient proliferation of these B cells probably due to high tonic BCR signaling. By this mechanism, IgG-tg B cells may produce specific antibodies without generating BCR signaling.

Ligand-induced activation of signaling molecules is generally thought to define the signaling function of receptors. However, our findings on BCR in this study suggest that ligand-independent basal signaling, or tonic signaling, plays a crucial role in signaling function thereby regulating cell activation. As a consequence, ligand-dependent signaling may not necessarily represent receptor function. The role of tonic signaling in the function of receptors other than BCR should be elucidated in the future.

### “Idling” Model for B Lymphocyte Activation

Studies on signaling function on IgG-BCR in primary B cells including our present study have demonstrated that IgG-BCR transmits a distinct signaling although the signaling properties of IgG-BCR in primary B cells are still controversial [17], [19]. Knock-in B cells in which the C region of IgM is replaced by that of IgG or those in which the cytoplasmic tail of IgM is replaced by that of IgG exhibit augmented Ca^2+^ signaling upon BCR ligation. In contrast, both MAPK activation and BCR-mediated gene expression were reduced in these knock-in B cells. What causes the discrepancy between Ca^2+^ signaling and other signaling pathways including MAPK activation is not yet known. However, depressed BCR ligation-induced signaling may be due to augmented tonic signaling through IgG-BCR. As BCR ligation-induced signaling is not completely abrogated in these knock-in mice, tonic signaling in these knock-in B cells may not be so high as in IgG-tg B cells in our study, and mildly increased tonic signaling may allow these knock-in B cells to generate BCR signaling to some extent. The Ig V genes are fixed in our IgG-tg B cells, whereas variety of V genes can be used in these knock-in B cells. In IgG knock-in B cells, VH genes that generate less tonic signaling may be selected. This assumption is supported by the finding that surface IgG expression is only mildly down-modulated in these knock-in mice [17], [19] compared to IgG-transgenic mice including our expressing VH-fixed IgG [23], [41], [42].

Although very high tonic BCR signaling may cause BCR signal defect upon BCR ligation, studies on CD22-deficient B cells suggest that such high tonic BCR signaling may not necessarily be required for augmented response to co-stimulation. B cells deficient in CD22, a negative regulator of BCR signaling, appear to generate increased tonic BCR signaling because of reduced surface BCR expression [43]–[46]. In these B cells, BCR ligation-induced signaling is rather augmented, suggesting that tonic signaling is not high enough to down-regulate ligation-induced BCR signaling. However, CD22-deficient B cells are hyper-responsive to CD40 ligation [47], [48], and generate augmented Ab response [49]. Thus, mildly increased tonic signaling may augment reactivity to T cell help in both CD22-deficient B cells and IgG tail knock-in B cells. Horikawa et al. [17] proposed “less-is-more” hypothesis in which less BCR-mediated gene expression is involved in augmented response of IgG^+^ B cells. Here we propose an alternative “idling” model in which high tonic signaling induces augmented B cell response by “idling” B cells so that they become ready to respond to co-stimulation.

The present study suggests that tonic BCR signaling regulates B cell response to antigen and T cell help. High tonic IgG-BCR signaling may be involved in augmented Ab production from IgG^+^ memory B cells, although BCR signaling properties of memory B cells is not yet well understood. High tonic IgG-BCR signaling may reflect strong BCR signaling capacity of IgG-BCR. However, molecular basis for efficient BCR signaling through IgG-BCR is not yet known. Previously, we stimulated various B cell lines with antigens, and demonstrated that IgG-BCR signaling is only weakly regulated by CD22 compared to IgM-BCR signaling [15]. In contrast, recent studies using IgG knock-in mice showed that CD22 regulates IgG-BCR signaling as well as IgM-BCR signaling generated by treatment with anti-Ig Ab. This discrepancy may be caused by difference in cells, reagents for BCR ligation or both [15], [17], [18], [19]. B cell lines appear to be resistant to tonic signaling-induced BCR signal regulation, and antigens induce stronger CD22-mediated signal regulation than anti-Ig Abs [25]. Recently, Engels et al. [50] demonstrated using B cell lines that tyrosine residue at the cytoplasmic tail of the membrane form of IgG is phosphorylated upon BCR ligation, and recruits Grb2 thereby augmenting BCR signaling. Multiple mechanisms may be involved in strong signaling capacity of IgG-BCR, and further studies are required to elucidate the molecular mechanisms for determining the level of tonic BCR signaling.

## Materials and Methods

### Ethics Statement

All experiments were approved by the animal committee of Tokyo Medical and Dental University.

### Mice

The transgene construct pIgG2a-NP encoding anti-NP Ig γ2a chain was generated by inserting a 4kb EcoRI fragment of pSVneoG2am (a kind gift of Dr. Reth) containing the rearranged V_H_186.2 gene segment into EcoRI-digested p1121 containing the Cγ2a gene [23] (a kind gift of Dr. Eibel). A 21-kb PvuI-KpnI fragment of pIgG2a-NP and a 12-kb PvuI-XhoI fragment of pSV-Vμ1 [51] that encodes anti-NP Ig μ chain were injected into fertilized C57BL/6 eggs. The transgenic mice expressing anti-NP Ig μ and γ2a chains were crossed with Ig κ chain-deficient mice [24], resulting in IgM-tg and IgG-tg mice. The amino acid sequence of the cytoplasmic tail of Ig μ chain and that of Ig γ2a chain are KVK and KVKWIFSSVVELKQTISPDYRNMIGQGA, respectively [52]. Xid mice (a gift of Dr. Takatsu) are described previously [31], [32]. All the mice were maintained in our animal facility under SPF conditions.

### Adoptive Transfer and Immunization

Spleen cells were prepared from either IgM-tg or IgG-tg mice, and erythrocytes were lysed by incubation with a solution consisting of 0.15 M NH_4_Cl, 10 mM KHCO_3_ and 0.1 mM EDTA, pH7.2. Cells were stained with PE-conjugated anti-CD23 Ab (BD Pharmingen) followed by incubation with anti-PE microbeads (Miltenyi Biotec), and CD23^+^ follicular B cells were purified using an autoMACS (Miltenyi Biotec). The purity of CD23^+^ cells was determined by flow cytometry using a LSR (Becton Dickinson) (purity >95%). In some experiments, cells were labeled with 5 µM CFSE (Molecular Probes) as described previously [53]. For analyzing the T cell-dependent response, Ly5-congenic C57BL/6 (B6.Ly5.1) [54] mice were treated intraperitoneally with 100 µg chicken γ-globulin (CGG) in CFA. After 7 days, mice were injected intravenously with 4×10^5^ purified CD23^+^ cells. One day later, recipients were immunized intraperitoneally with 50 µg NP_25_-CGG in alum. For analyzing the T cell-independent response, Ly5-congenic C57BL/6 (B6.Ly5.1) mice were injected intravenously with 4×10^5^ purified CD23^+^ cells. One day later, recipients were immunized intravenously with 50 µg 4-hydroxy-3-nitrophenyl acetyl (NP)- conjugated Ficoll (NP_50_-Ficoll) in PBS.

### ELISA and ELISPOT

Serum anti-NP Ab titers were measured by an ELISA using 96-well plates coated with NP-BSA and anti-mouse Ig Ab specific to Ig M, IgG2a or λ L chain (Southern Biotechnology Associates). Splenic Ab-forming cells (AFCs) were measured by standard ELISPOT assay as described previously [55]. In brief, MultiScreen-HA 96-well plates (Millipore) were coated with 50 µg/ml anti-λ Ab (Southern Biotechnologies). Spleen cells were distributed to the plates and were incubated for 5 h at 37°C in a CO_2_ incubator. Plates were incubated with biotin-labeled anti-IgM^a^ Ab and anti-IgG2a^a^ Ab followed by reaction with alkaline phosphatase-labeled streptavidin (BD PharMingen).

### Flow Cytometry

Spleen cells were incubated with anti-low-affinity Fc receptor for IgG (FcγRII/III) Ab (2.4G2), and were stained with the following reagents: biotin-labeled anti-CD43 Ab, FITC-labeled anti-IgM Ab (Southern Biotechnologies), FITC- and Alexa Fluor 647-labeled anti-B220 Ab, FITC-labeled anti-CD3 Ab (eBioscience), PE-labeled anti-CD23 Ab (eBioscience), FITC-labeled anti-CD21 Ab (eBioscience), biotin-labeled anti-IgG2a Ab (Southern Biotechnologies), biotin-labeled anti-CD40 Ab (BD PharMingen), FITC-labeled anti-CD22 Ab, biotin-labeled anti-CD19 Ab (BD PharMingen), biotin-labeled anti-MHC class II Ab (BD PharMingen), PE-conjugated anti-CD86 Ab (BD PharMingen), Alexa Fluor 647-labeled anti-Fas Ab, biotin-labeled anti-CD93 Ab (BD PharMingen), PE-conjugated anti-mouse/human CD44 (eBioscience), FITC- and biotin-labeled anti-Ly5.2 Ab, PE-conjugated anti-CD38 Ab (BD PharMingen), 4-hydroxy-3-iodo-5-nitrophenyl acetyl-conjugated phycoerythrin (NIP-PE), Biotin-labeled Abs were visualized by PerCP-conjugated streptavidin (BD PharMingen). 4, 6-diamino-2-phenylindole (DAPI) (SIGMA) was used for exclusion of dead cells. Cells were analyzed using a LSR with CELLQuest™ software (Becton Dickinson).

### Immunohistochemical Analysis

Spleens were embedded in Tissue-Tek O.C.T. compound (Sakura, The Netherlands), snap-frozen in liquid nitrogen and stored at −80°C. Cryostat sections (7-µm thick) were mounted onto slide glass, air dried, and fixed in acetone for 20 min at room temperature. The sections were incubated with blocking buffer (PBS containing 0.5% BSA and 0.05% NaN_3_) containing anti-FcγRII/III Ab (2.4G2) for 30 min, and were stained at room temperature for 60 min with PE-conjugated anti-CD38 Ab, and either biotin-labeled anti-IgM^a^ Ab/anti-IgM or biotin-labeled anti-IgG2a Ab, followed by reaction with FITC-labeled streptavidin (DAKO). The sections were analyzed with a confocal laser-scanning microscope LSM510 (Carl Zeiss).

### Cell Culture

Cells were labeled with 2 µM CFSE (Molecular Probes) as described previously [49], [54]. CFSE-labeled or unlabeled cells were cultured in RPMI 1640 medium supplemented with 10% FCS, 50 µM 2-ME, and 1mM glutamine. Purified CD23^+^ follicular B cells (1∼2×10^5^) were cultured in 200 µl of the culture medium with 0.2 µg/ml of the antigen NP_15_-BSA, 10 µg/ml anti-CD40 mAb FGK45 or both. Alternatively, cells were cultured with 10 µg/ml anti-IgM F(ab′)_2_, anti-IgG(H+L) F(ab′)_2_ or anti-λ L chain Ab. Cells were collected, and the percentages of apoptotic cells were estimated by flow cytometric measurement of cells with hypodiploid DNA. For assessing cell cycling, cells were pulsed with 20 µM 5-bromo-2′-deoxyuridine (BrdU) for the last 20 min of culture, and then fixed with 70% ethanol overnight at −20°C. Cells were stained with FITC-labeled anti-BrdU Ab (BD PharMingen) and propidium iodide, and analyzed by flow cytometry using a FACSCalibur (BD PharMingen). CFSE-labeled cells were analyzed by flow cytometry for CFSE fluorescence.

### Western Blotting

Purified CD23^+^ follicular splenic B cells were stimulated with 0.2 µg/ml NP_15_-coupled BSA (NP-BSA). Cells were lysed in Trinton X-100 lysis buffer (1% Triton X-100, 10% glycerol, 150 mM NaCl, 2 mM EDTA, 0.02% NaN_3_, 10 µg/ml PMSF, and 1 mM Na_3_VO_4_). Total cell lysates were separated on SDS-PAGE and transferred to polyvinylidene difluoride membranes. Membranes were incubated with peroxidase-conjugated anti-phosphotyrosine mAb 4G10 (Upstate Biotechnology). Alternatively, membranes were reacted with rabbit anti-mouse phospho-ERK Ab (New England Biolabs), or followed by peroxidase-conjugated anti-rabbit IgG Ab (New England Biolabs), or anti-β-tubulin mAb TUB 2.1 (Seikagaku Kogyo) followed by reaction with peroxidase-conjugated anti-mouse IgG Ab (Amersham Biosciences). Proteins were then visualized with an ECL system (Amersham Biosciences).

### BCR-Induced Calcium Flux

Purified CD23^+^ follicular B cells were incubated in culture medium containing 5 µM Fluo-4/AM (Molecular probes) at 37°C for 30min. Cells were stimulated with 0.2 µg/ml NP_15_-BSA and Fluo-4 fluorescence was measured continuously by flow cytometry using a LSR (Becton Dickinson).
